# FBXW7-mediated CHK2 regulation modulates DNA damage response and cellular stability in Huntington’s disease

**DOI:** 10.1038/s41420-025-02798-x

**Published:** 2025-11-03

**Authors:** Tae Eun Kang, Yu Min Lee, Seung Ho Choi, KyoungJoo Cho

**Affiliations:** 1 Department of Life Science, Kyonggi University, Suwon, South Korea; 2 Department of Health Sciences and Technology, Samsung Advanced Institute for Health Sciences and Technology, Sungkyunkwan University, Seoul, Korea; 3 Samsung Biomedical Research Institute, Research Institute for Future Medicine, Samsung Medical Center, Seoul, Korea

**Keywords:** Huntington's disease, Huntington's disease, DNA damage response

## Abstract

DNA damage activates the DNA damage response (DDR) machinery. However, aging impairs DDR in neurons, thereby increasing susceptibility to neurodegenerative diseases, such as Huntington’s disease (HD). The mutant huntingtin (mHTT) protein interferes with DNA repair, leading to DNA lesions and a feedback loop of cellular stress that accelerates neurodegeneration. Although the individual roles of FBXW7, ATM, and checkpoint kinase (CHK) are well-known in DDR, their combined roles in HD remain unclear. In this study, we investigated the FBXW7-mediated CHK2 pathway in HD, in which mHTT levels increase, whereas wild-type (WT) HTT levels decrease. HD cells containing mHTT or expanded polyQ-HTT were more prone to DNA damage than cells containing wtHTT or normal-length polyQ, demonstrating the increased vulnerability of HD neurons. Downregulating the expression of FBXW7 reduces susceptibility to DNA damage and promotes cellular stability. Additionally, FBXW7 specifically prevented CHK2 degradation, but not CHK1 degradation. This suggests a selective role in DDR regulation. Thus, the FBXW7-CHK2 pathway may alleviate DNA damage in HD by supporting DDR and inducing cell cycle arrest. The intricate relationship between DDR and HTT is fundamental to the pathophysiology of HD. Elucidating these mechanisms could facilitate the development of new therapeutic strategies that enhance DNA repair or correct DDR dysfunction, thereby slowing disease progression or delaying symptom onset. Understanding this pathway may provide insights into the targeting of DNA repair defects in HD and related neurodegenerative disorders.

## Introduction

DNA damage activates the DNA damage response (DDR) machinery, which is essential for genomic integrity. Although the precise mechanisms underlying Huntington’s disease (HD) remain unclear, accumulating evidence suggests that impaired DDR, particularly involving double-strand break (DSB) repair, may contribute to HD pathogenesis alongside other disrupted cellular pathways. DDR weakening in the aged brain and HD neurons causes genomic instability [1, 2]. Genetic modifiers of HD are linked to DNA repair and mitochondrial function in both symptomatic and asymptomatic patients with HD [2, 3]. The mutant Huntingtin protein, with expanded CAG repeats, forms toxic fragments that accumulate in neurons and disrupt normal function [2, 4]. The mHTT interferes with DNA repair, consequently promoting the formation of DNA lesions and exacerbating neurodegeneration through a feedback loop of cellular stress [2, 5]. Unrepaired DNA DSBs prolong DDR activation. Given the essential role of DSB repair pathways, such as homologous recombination (HR) and nonhomologous end joining in maintaining genome integrity, we investigated how HTT depletion affects the cellular response to DSBs and other forms of DNA damage [2]. Senescent neurons exhibit metabolic dysregulation, mitochondrial impairment, and increased secretion of prooxidant and proinflammatory factors that contribute to age-related neurodegeneration [6]. The MRN complex, which is composed of Mre11, Rad50, and Nbs1, detects DNA breaks and activates the ATM kinase, which phosphorylates and activates Checkpoint kinase protein, including Checkpoint kinase 2 (CHK2) [7]. Activated CHK2 stabilizes and activates p53 through phosphorylation, thereby coordinating DNA repair and apoptosis [7, 8]. CHK2 degradation occurs via the ubiquitin-proteasome pathway, and the CRL E3 ubiquitin ligase complex is mainly charged [7]. Among them, the role of FBXW7 has been mentioned in neuronal disease [9, 10], but the exact role of FBXW7 in this process (DDR of neurodegenerative diseases) remains largely understudied [10].

FBXW7, an F-box and WD repeat domain-containing protein, is a substrate recognition subunit of the SKP1-Cullin1-F-box E3 ubiquitin ligase complex responsible for targeted protein degradation [9]. In the brain, FBXW7 primarily modulates neurodevelopment via substrates, such as Notch and c-Jun, the stability of which is controlled by FBXW7-mediated ubiquitination [11]. Although FBXW7 mutations are not linked to neurodevelopmental disorders, substrate mutations have been implicated in related diseases [10]. In Alzheimer’s disease, FBXW7 may regulate cellular senescence by promoting telomere uncapping through the degradation of the telomere protection protein TPP1 [10, 12]. In HD, FBXW7 also contributes to the pathogenesis of HD by targeting p53 for degradation. p53 interacts with dynamin-related protein 1 to promote mitochondrial fragmentation, a key mechanism involved in HD neurodegeneration [13, 14].

We hypothesized that mHTT disrupts the FBXW7-mediated DDR and accelerates neuronal damage. Our study showed that FBXW7 primarily blocks CHK2 degradation, but not that of CHK1, in HD cells. The subsequent increase in the expression of Ku70, a DNA repair protein, may delay HD pathology. Further investigation of the FBXW7-CHK2 axis in HD cells revealed a novel link in mHTT-induced DDR dysfunction, consequently providing potential therapeutic targets.

## Results

### HD neuronal cells containing expanded polyQ are susceptible to DNA damage

**Fig. 1:**
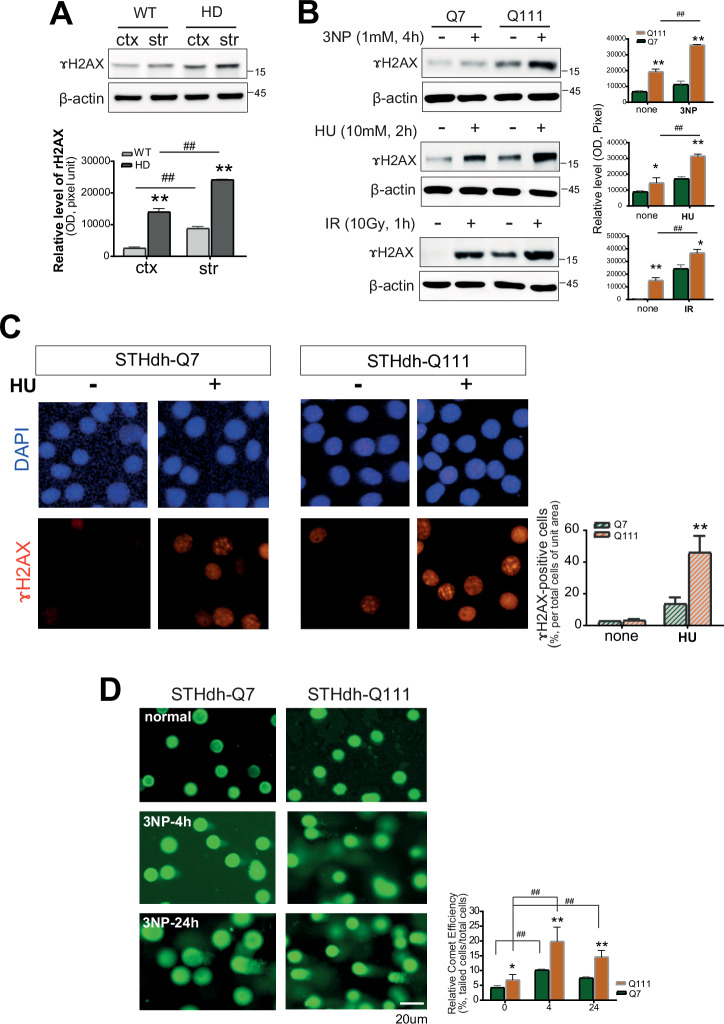
HD neurons with expanded polyQ repeats show increased DNA damage. **A** γH2AX levels are elevated in symptomatic R6/2 HD mouse brains (*n* = 5 each) in Western blot analysis. The quantitative values were normalized to each beta-actin. **B** mHTT (Q111) STHdh cells show increased γH2AX after DNA damage in Western blot analysis (*n* = 7). The quantitative values were normalized to each beta-actin. **C** Immunocytochemistry presents Q111 cells have more γH2AX foci after HU exposure (*n* = 5). **D** Comet assay shows more DNA breaks in Q111 cells treated with 3NP (*n* = 5). All experiments were independently performed at least three times to ensure reproducibility. Data are expressed as the mean ± std from at least three independent experiments. Statistical significance was determined using t-test; *p < 0.01, **p < 0.001, versus WT cells; ^*#*^*p* < 0.01, ^*##*^*p* < 0.001, between the same group. *, indicating statistical significance among time points within each group (siNC or siHTT). #, indicating statistical significance between siNC and siHTT groups at a time-point.

We characterized the response to DNA damage in normal and HD neuronal cells expressing mHTT with expanded polyQ repeats. We evaluated γH2AX, the phosphorylated form of the histone variant H2AX, was used as a biomarker for DNA DSBs. γH2AX expression increased in the brain tissues of symptomatic 12-week-old R6/2 HD mice compared to that in wild-type (WT) mice. Furthermore, γH2AX expression in HD mice was slightly higher in the striatum than in the cortex (Fig. 1A). STHdh HD cells containing expanded polyQ (Q111) also showed increased γH2AX levels compared to WT (Q7) cells. In this study, mouse-derived STHdh Q7 and Q111 cells served as a physiologically relevant model system that endogenously expresses WT or mutant HTT, respectively, allowing us to validate our findings in a genetically accurate HD setting. Moreover, γH2AX levels were significantly elevated upon exposure to DNA-damaging agents, such as ionizing radiation (IR), hydroxyurea (HU), or mitochondrial toxin 3-nitropropionic acid (3NP), indicating increased DNA damage (Fig. 1B). HD neuronal cells consistently exhibited significantly higher levels of DNA damage upon exposure to HU for mimicking aged condition and leading proliferation DNA damage, as quantified based on γH2AX foci (Fig. 1C). The immunocytochemical staining of STHdh HD cells (Q111) revealed a significantly higher number of γH2AX-positive cells with distinct foci formation than in STHdh WT cells (Q7). This demonstrates reduced cell viability compared to that in normal neuronal cells. Comet assays were subsequently performed using WT (Q7) and HD (Q111) cells by 3NP exposure (Fig. 1D). Consistent with the γH2AX results in WB or ICC, DNA damage was observed in both cell types. Analysis of comet tails upon 3NP exposure revealed increased DNA damage in both Q7 and Q111 cells at 4 h, which persisted for 24 h. The increase in DNA damage was more pronounced in Q111 cells than in Q7 cells (Fig. 1D, right graph). These results demonstrate that HD neuronal cells with expanded polyQ repeats increased γH2AX expression and comet tail length, which exhibit elevated DNA damage. It showed greater susceptibility to DNA damage in the striatum than in the cortex.

### Reduction of wtHTT expression by increasing expanded polyQ-HTT or siHTT causes susceptibility to DNA damage

**Fig. 2:**
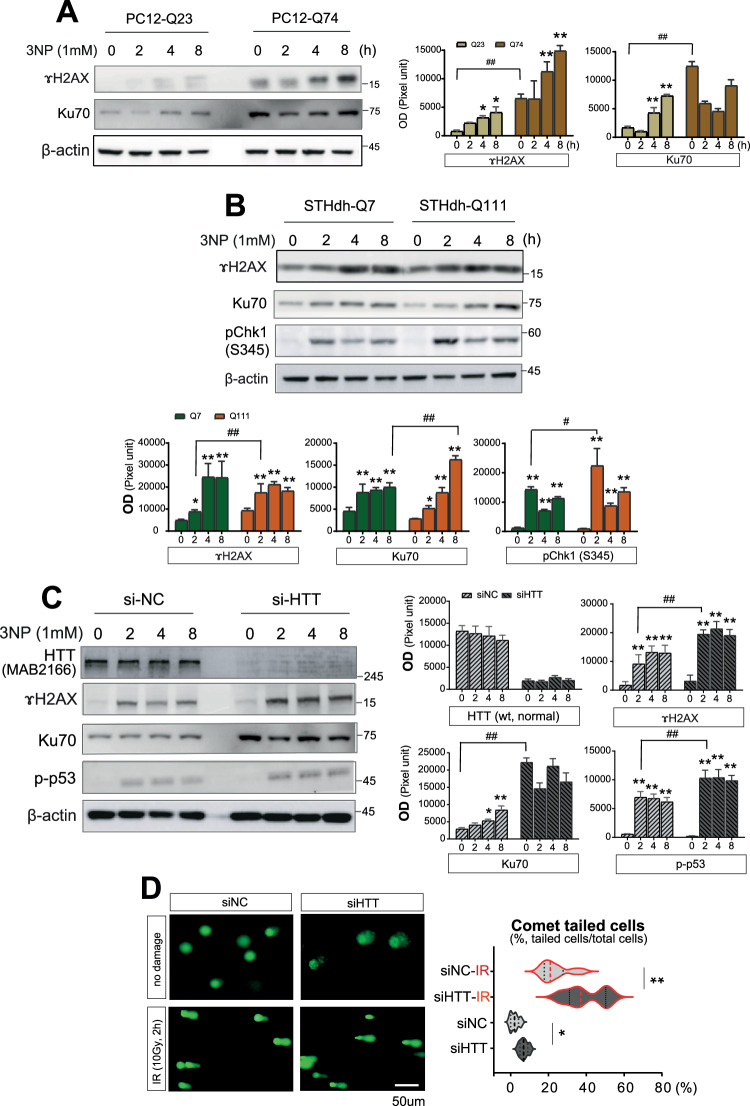
Mutant HTT affects DDR and DNA damage. **A** Western blot analysis shows polyQ74-PC12 cells increased γH2AX and Ku70 after 3NP (*n* = 5). The quantitative values were normalized to each beta-actin. **B** Q111-STHdh cells have earlier, stronger γH2AX, elevated Ku70, and higher CHK1 (Ser345) after 3NP by Western blot analysis (*n* = 7). The quantitative values were normalized to each beta-actin. **C** wtHTT knockdown in HeLa cells increases γH2AX and Ku70 and activates p53 after 3NP (*n* = 5). The quantitative values were normalized to each beta-actin. **D** wtHTT-depleted HeLa cells show increased comet tail length and are more sensitive to IR (*n* = 5). All experiments were independently performed at least three times to ensure reproducibility. Data are expressed as the mean ± std. Statistical significance was determined using one-way analysis of variance, followed by Tukey’s post-hoc test. *p < 0.01, **p < 0.001; ^*#*^*p* < 0.01, ^*##*^*p* < 0.001, t test between the same group. *, indicating statistical significance among time points within each group (siNC or siHTT). #, indicating statistical significance between siNC and siHTT groups at a time-point.

To assess how polyglutamine expansion influences DNA damage sensitivity and cellular stress responses, we validated the impaired DDR in the PC12 cells stably expressing either WT HTT (Q23) or expanded polyQ HTT (Q74). PC12 cells conditionally expressing polyQ23 (nonpathogenic) or polyQ74 (pathogenic) HTT in response to doxycycline were used to investigate the effects of mHTT on the DDR. γH2AX levels increased in a time-dependent manner following 3NP exposure. This increase was significantly more pronounced in cells expressing polyQ74 than in those expressing polyQ23 (Fig. 2A). Ku70 expression was also markedly elevated in polyQ74-expressing cells, suggesting that mHTT enhanced DDR activation. It demonstrates that mHTT, including abnormally expanded polyQ alone, is sufficient to induce the condition of DNA damage even in the absence of external stress. To further validate the role of mHTT in the modulation of the DDR, we used the STHdh cell model of HD, and γH2AX levels increased in both Q7 and Q111 cells following 3NP exposure; however, Q111 cells exhibited an earlier onset at 2 h and greater magnitude of γH2AX induction than Q7 cells (Fig. 2B). Similarly, Ku70 expression was significantly upregulated in Q111 cells 8 h post-treatment, indicating stronger DDR activation. Moreover, phosphorylated CHK1 (pCHK1, Ser345), a key mediator of cell cycle arrest upon DNA damage, was markedly elevated at 2 h in Q111 cells compared to Q7 cells, suggesting that mHTT accelerates the cellular recognition of DNA damage and activation of downstream signaling pathways. We employed siRNA-mediated knockdown of wtHTT in HeLa cells to determine whether the loss of normal huntingtin (wtHTT), which has been implicated in the regulation of genomic stability, affects DDR. Western blotting revealed a marked increase in γH2AX levels following wtHTT depletion, consistent with the response observed in the expanded polyQ mHTT expression (Fig. 2C). The expression of Ku70, a critical DNA repair factor, was significantly upregulated in wtHTT-deficient cells following DNA damage. Additionally, phosph-p53, a key transcriptional regulator that is activated in response to genotoxic stress, showed more robust activation in wtHTT-knockdown cells than in controls. To assess the extent of DNA damage associated with altered DDR signaling, we performed comet assays to visualize and quantify the DNA strand breaks in cells with reduced wtHTT expression. Cells in which wtHTT expression was downregulated by siRNA exhibited longer comet tails under basal conditions, indicating increased endogenous DNA damage (Fig. 2D, the upper images). DNA damage was further exacerbated upon exposure to IR (Fig. 2D, the lower images). Quantitative analysis confirmed significantly greater DNA damage in wtHTT-deficient cells than in controls, both at baseline and after IR. These findings indicate that susceptibility to DNA damage was enhanced by not only increased mHTT but also loss of wtHTT, including normal length polyQ stretch.

### FBXW7 interacted with wtHTT, and an FBXW7-induced decrease in CHK2 levels was associated with DDR in HD cells

**Fig. 3:**
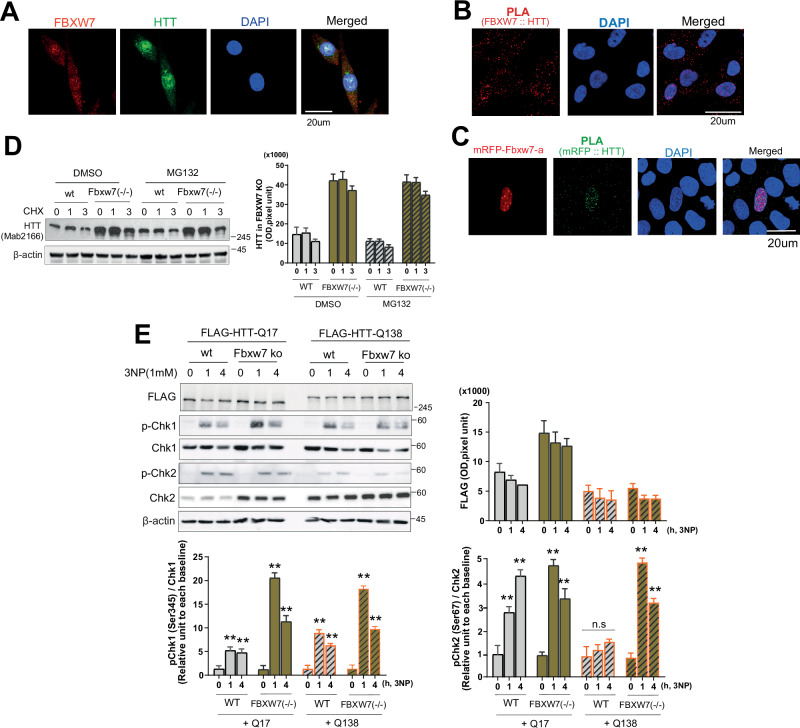
Increased FBXW7, lower CHK2 link to DDR in HD. **A** Double immunocytochemistry between FBXW7 and HTT displays co-localized in the same cells in HeLa cells, indicating relationship between two molecules in cells. **B** PLA shows FBXW7 (Abcam, Ab192328, anti-rabbit) interacts with wtHTT (Millipore, Mab2166, anti-mouse) in HeLa cells (*n* = 5). **C** PLA with mRFP-FBXW7 (RFP, Proteintech, anti-rabbit) and wtHTT (Millipore, Mab2166, anti-mouse) shows specific interaction with FBXW7 and wtHTT (*n* = 5). **D** Western blot analysis presents that wtHTT levels are higher in FBXW7−/− HCT116 cells by cycloheximide chasing (*n* = 7). The quantitative values were normalized to each beta-actin. **E** In FBXW7−/− cells, pCHK1 and pCHK2 increase after 3NP, but CHK2 activation is lower, and total CHK2 increases (*n* = 5). The quantitative values were normalized to each beta-actin. All experiments were independently performed at least three times to ensure reproducibility. Data are expressed as the mean ± std. Statistical significance was determined using one-way analysis of variance, followed by Tukey’s post-hoc test. *p < 0.01, **p < 0.001.

We identified FBXW7 as a candidate molecule involved in both the DDR and in HTT degradation. RNAseq analysis of brain tissues from R6/2 HD mice revealed a significant increase in FBXW7 expression after symptom onset compared with pre-symptomatic stages (GEO accession number: GSE276407). Primarily, double immunocytochemistry of wtHTT and FBXW7 displayed co-localization of two molecules (Fig. 3A). To more validating interaction, PLA was performed. Distinct red fluorescent puncta were observed, indicating direct interaction between endogenous FBXW7 and wtHTT (Fig. 3B). This displays that FBXW7 modulates HTT stability or function through direct physical association. We performed PLA in HeLa cells transiently expressing mRFP-tagged FBXW7 (α isoform) to investigate the specificity of the interaction between FBXW7 and wtHTT. Distinct green-fluorescent puncta of PLA were exclusively detected in cells expressing mRFP-FBXW7, confirming a direct and selective interaction between FBXW7 and wtHTT (Fig. 3C). Contrastingly, no PLA signals were detected in cells lacking mRFP-FBXW7, supporting the specificity of this interaction. To investigate the regulatory mechanisms controlling wtHTT levels, we primarily examined its stability in normal human colorectal carcinoma HCT116 cells. HCT116-FBXW7 knockout cells provided a well-defined genetic model to examine the consequences of FBXW7 loss on DDR regulation independently of mutant HTT. The basal levels of wtHTT were significantly higher in FBXW7−/− cells than in normal HCT116 cells (Fig. 3D). However, MG132 treatment of FBXW7−/− cells did not substantially increase wtHTT levels despite elevated basal levels (Fig. 3D).

FBXW7 could regulate wtHTT through indirect pathways, and we investigated the role of FBXW7 in wtHTT function in the context of DDR. We exposed FBXW7−/− cells expressing either wtHTT (polyQ17) or mHTT (polyQ138) to 3NP to further assess the role of FBXW7 in DDR (Fig. 3E). Phosphorylated CHK1 (pCHK1, Ser345) and CHK2 (pCHK2, Thr68) levels were elevated following DNA damage; however, the magnitude of CHK2 activation was markedly lower than that of CHK1 activation. Under HTT-Q17 (normal condition), the expression of CHK1 did not show significant changes over time following DNA damage induced by 3NP, and this pattern was consistent in both wt cells and FBXW7−/− cells. In contrast, under HTT-Q138 (HD-like conditions), CHK1 expression showed a time-dependent decrease in both WT and FBXW7−/− cells, although basal levels were comparable between the two groups. In the case of CHK2, a distinct pattern was observed compared to CHK1. CHK2 expression did not change over time after 3NP treatment in any group. However, under HTT-Q17 conditions, basal CHK2 expression was markedly elevated in FBXW7−/− cells compared with WT. Under HTT-Q138 conditions, basal CHK2 levels were higher than those observed under HTT-Q17, and this increase was further pronounced in FBXW7−/− cells compared to WT. Furthermore, as shown in the graphs (Fig. 3E, lower), when normalized to total CHK1, the pattern of pCHK1 was comparable across all conditions, showing no significant differences between cells expressing normal HTT (Flag-Q17) or mutant HTT (expanded Flag-Q138), and this pattern remained consistent regardless of FBXW7 expression status. In contrast, the pCHK2, normalized to total CHK2, exhibited a distinct response. In cells expressing normal HTT (Flag-Q17), pCHK2 was activated in a similar manner to that of pCHK1 following DNA damage. However, in cells expressing mutant HTT (Flag-Q138), pCHK2 failed to be activated in response to DNA damage. Interestingly, when FBXW7 was knocked down, cells expressing mutant HTT regained the ability to activate pCHK2 upon DNA damage, displaying a response pattern comparable to that observed in the Flag-Q17 condition. Given the results, FBXW7 directly interacts with wtHTT and negatively regulates CHK2 amounts, which implies a higher level of FBXW7 involves in wtHTT regulation and impaired DDR.

### FBXW7 selectively regulates CHK2 expression, and downregulation of FBXW7 expression ameliorated susceptibility of HD cells to DNA damage

**Fig. 4:**
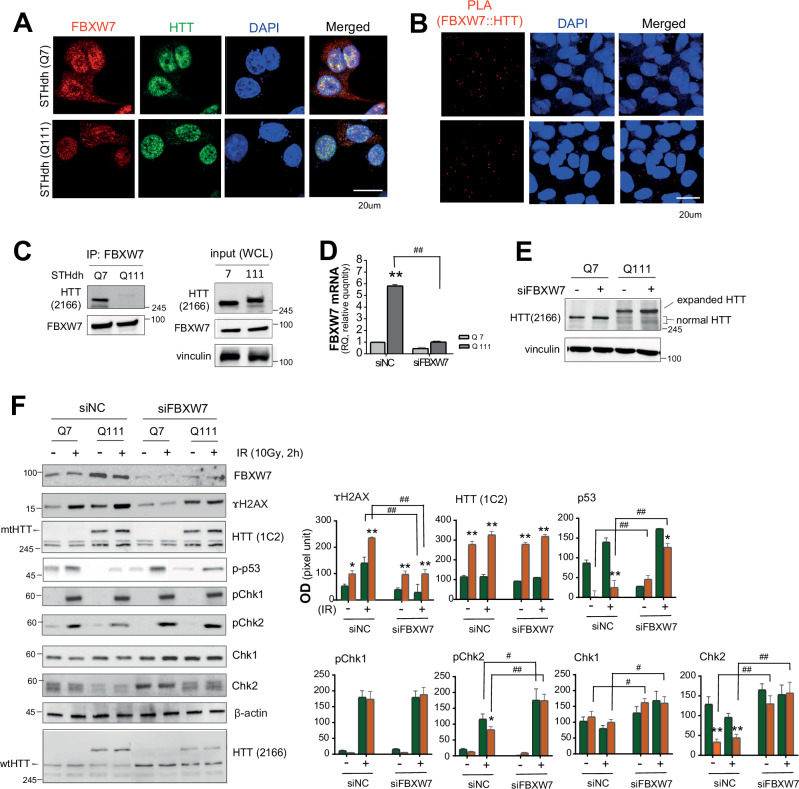
FBXW7 regulates CHK2, affects DDR in STHdh-HD cells. **A** Double immunocytochemistry between FBXW7 and HTT displays co-localized in the same cells in HD cells, STHdh (Q7) and STHdh (Q111). It indicates relationship between two molecules in HD cells. **B** PLA between FBXW7 (Abcam, Ab192328, anti-rabbit) and HTT (Millipore, Mab2166, anti-mouse) indicates interaction between FBXW7 and HTT in STHdh HD cells (*n* = 5). **C** Co-immunoprecipitation showed a specific interaction between FBXW7 and wtHTT. **D** The qPCR analysis showed that FBXW7 mRNA level was markedly higher in STHdh-Q111 cells than Q7 cells and was effectively downregulated by siFBXW7 treatment. (*n* = 5). **E** Under downregulating by siFBXW7, total HTT protein amounts, including wtHTT amounts, were significantly increased in STHdh HD cells. **F** Western blot shows γH2AX, p53, CHK2 changes in Q17/Q111 cells with IR and siFBXW7 (*n* = 5). FBXW7 knockdown differentially modulated DDR signaling in HD cells, suppressing γH2AX induction while selectively enhancing p53 and CHK2 activation in Q111 cells, whereas CHK1 activation remained largely unaffected. The quantitative values were normalized to each beta-actin, and each was shown in the quantitative graph. All experiments were independently performed at least three times to ensure reproducibility. Data are expressed as the mean ± std. Statistical significance was determined using one-way analysis of variance, followed by Tukey’s post-hoc test. *p < 0.01, **p < 0.001; ^*#*^*p* < 0.01, ^*##*^*p* < 0.001, *t test* between the same group. *, indicating statistical significance among time points within each group (siNC or siHTT). #, indicating statistical significance between siNC and siHTT groups at a time-point.

To investigate the relationship between FBXW7 and HTT in HD cells, double immunocytochemistry was performed, and the result displayed the two molecules were co-localized (Fig. 4A). In addition, to examine endogenous protein–protein interaction in STHdh cells, we conducted PLA in STHdh (Q7 and Q111) HD cells. In both cells, red puncta of PLA were detected (Fig. 4B). As well as PLA, co-immunoprecipitation in STHdh cells showed the result confirming a specific interaction between FBXW7 and HTT, with a stronger association observed for wtHTT (Fig. 4C). To investigate the role of FBXW7 in HD pathology, we performed siRNA-mediated FBXW7 knockdown in STHdh cells, and the FBXW7 transcript was confirmed via qPCR (Fig. 4D). Additionally, downregulated FBXW7 increased wtHTT expression level in STHdh HD cells (Fig. 4E). Correspondingly, a differential response to IR-induced DNA damage was observed following γH2AX induction depending on FBXW7 expression levels and HTT genotype. γH2AX levels did not significantly increase in siFBXW7 both in Q7 or Q111 under none-damage condition, whereas DNA damage led to an obvious different pattern. Specifically, under siFBXW7, γH2AX level was rather significantly decreased to IR both in Q7 and Q111 (Fig. 4F). Moreover, p53 expression was upregulated in a damage-dependent manner and was unaffected by the downregulation of FBXW7 expression in Q17 cells. However, p53 activation in response to DNA damage in Q111 cells was minimal compared to that in Q17 cells, but was significantly enhanced upon the FBXW7 knockdown. A similar pattern was observed for CHK2 activation. CHK2 expression was low in Q111 cells compared to that in Q7 controls; however, it significantly increased upon the downregulation of FBXW7 expression. Contrastingly, FBXW7 knockdown slightly increased CHK1 levels; however, no substantial difference in CHK1 expression or activation was observed between Q7 and Q111 cells. Moreover, pCHK1 levels following DNA damage were comparable between Q7 and Q111 cells and remained unchanged following FBXW7 knockdown, indicating that FBXW7 did not selectively regulate CHK1 activation (Fig. 4F).

**Fig. 5:**
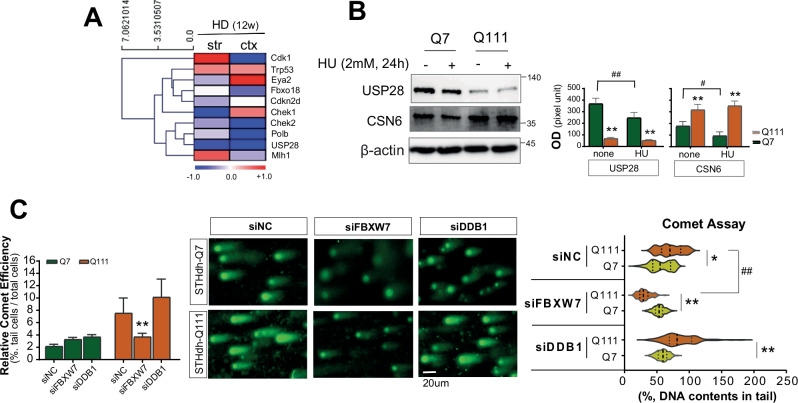
Altered USP28 and CSN6 expression in HD cells and modulation of DDR. **A** The heatmap of RNAseq from HD mice brain tissue was selected in DNA damage or DNA repair category. CHK2 and USP28 were downregulated in both striatal and cortical regions. **B** USP28 decreases, CSN6 increases in Q111 cells in Western blot analysis (*n* = 5). The quantitative values were normalized to each beta-actin. **C** Quantification of DNA damage using the comet assay including siFBXW7 or siDDB1 after 3NP (1 mM, 4 h) presents that 3NP affect DNA damage in Q7 and Q111 cells (*n* = 5). All experiments were independently performed at least three times to ensure reproducibility. Data are expressed as the mean ± std. Statistical significance was determined using a t-test analysis of variance. *p < 0.01, **p < 0.001, versus control; ^*#*^*p* < 0.01, ^*##*^*p* < 0.001, between the same group. *, indicating statistical significance among time points within each group (siNC or siHTT). #, indicating statistical significance between siNC and siHTT groups at a time-point.

Based on our previous RNAseq analysis (GSE276407, Fig. 5A), we further quantified USP28 and CSN6 expressions in HD model cells (Fig. 5B). The expression of USP28 was significantly decreased in Q111 mHTT-expressing cells compared to that in Q7 WT controls. In contrast, the expression of CSN6, which enhances the E3 ligase activity of FBXW7, was significantly upregulated in Q111 cells compared to that in Q7 cells (Fig. 5B). The quantification of DNA damage using the comet assay revealed corresponding changes in DNA integrity. Cells with comet tail were assessed in siRNA negative control (siNC), siFBXW7, and siDDB1, which is a DNA Damage Binding Protein 1, one of the main DDR pathway factors, exhibited significantly increased to 3NP exposure for 4 h. DNA damage was indicated by comet-tailed cells per total cells (Fig. 5C, the right graph). As an additional positive control, we compared the effects of DDB1 downregulation on protein degradation via DDR-associated ubiquitination. Exposure to 1 mM 3NP for 4 h led to an increase in the comet tail length, which was significantly greater in Q111 cells than in Q7 cells under siNC treatment (Fig. 5C, the middle). When siFBXW7 was treated, the comet tail length and moments were reduced than that of siNC treated cells just in Q111 cells. With siDDB1 treatment, although tail length was slightly shortened, it was not significant compared to that of siNC treatment. Furtherly, it remained significantly longer in Q111 cells than in Q7 cells. The quantification of comet was assessed by DNA contents in comet tail of each cell (Fig. 5C, the right graph). These results demonstrate that FBXW7 knockdown modulated DDR, suggesting the role of FBXW7 on mHTT instability via regulating DDR components.

### Downregulation of FBXW7 expression mitigated cellular stability in HD and ameliorated HD progression

**Fig. 6:**
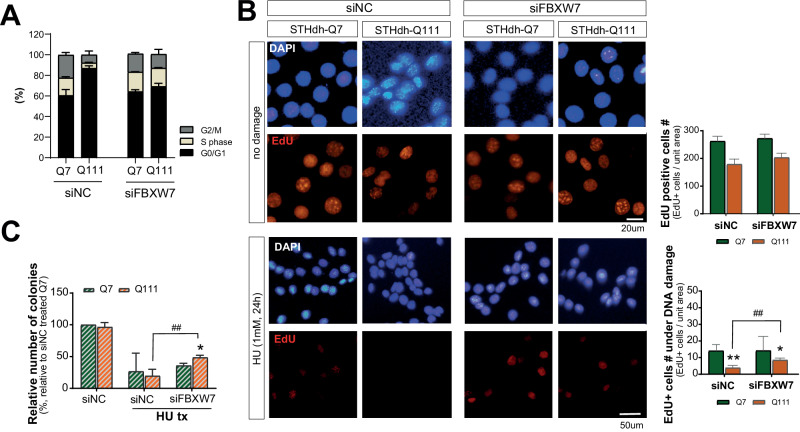
FBXW7 modulates DNA damage response in HD cells. **A** Cell cycle analysis shows Q111 cells show G1 arrest, alleviated by siFBXW7 (*n* = 5). **B** EdU staining: siFBXW7 promotes proliferation in Q111 cells after 3NP (*n* = 5). **C** Clonogenic assay indicates siFBXW7 improves Q111 cell recovery after HU (hydroxyurea), enhancing colony formation (*n* = 3). All experiments were independently performed at least three times to ensure reproducibility. Data are expressed as the mean ± std. Statistical significance was determined using a t-test analysis of variance. *p < 0.01, **p < 0.001, versus control; ^*#*^*p* < 0.01, ^*##*^*p* < 0.001, between the same group. *, indicating statistical significance among time points within each group (siNC or siHTT). #, indicating statistical significance between siNC and siHTT groups at a time-point.

The cell cycle pattern of HD (STHdh-Q111) cells showed G1 arrest, and a markedly shortened S phase compared to that of normal cells (Fig. 6A). FBXW7 knockdown alleviated the G1 arrest. EdU staining was subsequently performed, and the number of EdU-positive proliferating cells was significantly lower in Q111 cells than in Q7 cells (Fig. 6B). Moreover, no significant changes in the number of proliferating cells were observed in Q7 cells after siFBXW7 treatment. Contrastingly, Q111 cells showed a significant increase in EdU-positive cells following siFBXW7 treatment (Fig. 6B; right panel, quantitative graph). Proliferation was markedly reduced in both Q7 and Q111 cells under DNA damage compared to that in untreated controls. Although a minimal difference in the number of EdU-positive cells was observed between the siNC and siFBXW7 conditions in Q7 cells, Q111 cells exhibited a two-fold increase in EdU-positive cells upon FBXW7 knockdown (Fig. 6B). Finally, we evaluated the functional consequences of modulating FBXW7 levels on the susceptibility of HD cells to DNA damage. The clonogenic assay demonstrated that Q111 cells failed to recover following DNA damage, likely because of impaired DDR mechanisms. This result was consistent with the EdU staining data. However, a minimal but significant increase in colony formation was observed upon the downregulation of FBXW7 expression, suggesting the partial restoration of DDR and improved cell survival (Fig. 6C). These results showed that HD cells exhibited G1 arrest and reduced proliferation, indicating that FBXW7 suppression can alleviate mHTT-associated cell cycle defects and DDR impairment.

## Discussion

Aging increases neuronal vulnerability to minor deficiencies in DNA repair proteins. Age-related neurodegenerative diseases, such as HD are related to DDR. In this study, we investigated the role of the FBXW7-CHK2 pathway in regulating the DDR in both general and HD-specific contexts by using several cell lines. Our data indicate that HD models exhibit increased vulnerability to DNA damage induced by various insults, including tissue-specific factors, IR, HU, and 3NP (Fig. 1). This susceptibility appears to be linked to wtHTT, as the absence of wtHTT, either through expanded polyQ expression or siHTT treatment, phenocopies the DNA damage vulnerability observed in HD models (Fig. 2) as increment of γH2AX or Ku70. Elevated levels of γH2AX, a sensitive biomarker of DNA DSBs, were observed in HD tissues and cells in this study and previous reports [15]. γH2AX phosphorylation rapidly occurs at serine 139 upon DSB induction and recruits repair proteins, serving as a critical DDR component [16, 17]. Increased γH2AX foci indicate either increased DNA damage or defective repair. Additionally, persistent γH2AX suggests unresolved lesions potentially leading to cell cycle arrest or apoptosis [18]. Consequently, an observed increase in the intensity or number of γH2AX foci within cells or tissues is highly indicative of elevated genomic instability, reflecting either an increased burden of DNA damage or a deficiency in the cellular DNA repair machinery. Persistent γH2AX signaling can also suggest chronic DNA damage or unresolved lesions that may ultimately lead to cell cycle arrest, apoptosis, or the accumulation of mutations in HD tissue and cells. HD neuronal cells harboring mHTT displayed markedly increased susceptibility to DNA damage and impaired repair capacity, as demonstrated by comet assays in the present study. In our study, loss of wtHTT as well as accumulation of mHTT modulated DDR activation and DNA damage in our study (Fig. 2). These findings suggest that loss of wtHTT alone is sufficient to trigger DDR activation, further highlighting its protective role in maintaining genome integrity.

**Fig. 7:**
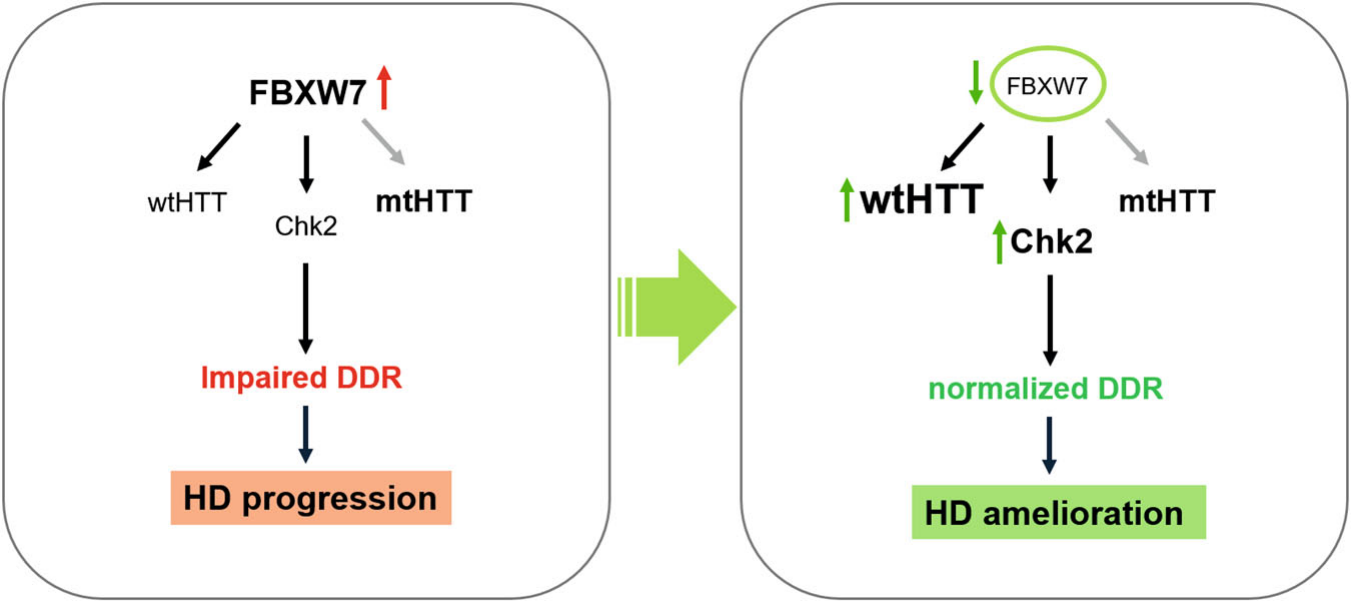
Schematic summary of FBXW7-mediated modulation of DDR and HD progression. In progressed HD cells, increased FBXW7 is negatively regulating wtHTT and CHK2 level. It leads reducing wtHTT stability and impairing DDR. Consequently, this cellular condition promotes disease progression (the left box). However, the current study suggests that FBXW7 downregulation restores CHK2 expression and DDR function, improves cell survival, and increases wtHTT levels, thereby contributing to HD amelioration (the right box).

Our findings indicated that impaired DDR in HD cells was driven by aberrantly elevated FBXW7 levels, leading to a subsequent decrease in CHK2 protein levels, as illustrated in the left panel of Fig. 7. Our proximity ligation assay revealed a direct and selective interaction between FBXW7 and wtHTT (Fig. 3), confirming the role of FBXW7 as a wtHTT-interacting protein. The dysregulation between FBXW7 and CHK2 leads to prolonged cell cycle arrest, increased susceptibility to DNA damage by showing extended comet tail, enhanced neuronal death, consequently exacerbating HD progression. FBXW7 mediates the degradation of key DDR regulators, including CHK1 and CHK2 [19]. In this study, however, CHK2 levels were significantly lower in HD cells than those in normal cells, whereas CHK1 levels remained stable. Additionally, reduction of FBXW7 expression restored CHK2, but not CHK1 levels (Fig. 3), suggesting that FBXW7 preferentially targets CHK2 for degradation in HD. FBXW7 KO with polyQ expression increased CHK2, but not CHK1 levels (Fig. 4). The downregulation of FBXW7 expression reduced DNA damage, likely by decreasing CHK2 ubiquitination and degradation [7, 19]. However, siFBXW7 improved the DDR and proliferation in HD cells (Q111) but not in WT cells (Q7) (Fig. 4). These findings support our hypothesis that FBXW7 regulates CHK2 in an HD-specific manner, thereby affecting cell cycle arrest and DDR progression. In general, it is mainly done by FBXW7 ubiquitinating CHK2 to promote its degradation [20]. It suppresses excessive activation of CHK2 in cancer cells [21]. Since abnormal accumulation of mHTT can induce cellular stress and DDRs, it may be expected that FBXW7 can alleviate these negative effects by mediating the degradation of mHTT. In our results, however, siFBXW7 involves in ameliorating DNA damage and protecting cellular stability or proliferation just in Q111 harboring STHdh cells not in Q7-STHdh. Our hypothesis was that FBXW7 E3 ligase degrades a molecule to regulate CHK2. While it has no specific role in normal cells, under HD context (Q111), CHK2 regulated by FBXW7 is presented in this study. In the HD context, it plays a role in interfering with the degradation of mHTT, exacerbating DNA damage, interfering with DDR reaction progression, re-entry into arrested cell cycle, or worsening the pathological symptoms resulting in Q111-HD cells. Additionally, USP28 and CSN6 expressions are differentially regulated in mHTT-expressing cells (Fig. 5). USP28 and CSN6 are key regulatory proteins involved in the DDR and protein stability pathways. USP28 is a deubiquitinase known to stabilize checkpoint proteins, such as CHK2, while CSN6 is a component of the COP9 signalosome complex that regulates protein degradation via the ubiquitin-proteasome system. It suggests that mHTT may contribute to altered FBXW7 regulation through opposing changes in USP28 and CSN6 expression, potentially shifting the balance toward increased FBXW7 activity.

After DNA damage, elevated pCHK1 levels in Q111 cells compared to Q7 cells suggest accelerated checkpoint activation, whereas stronger p53 activation in wtHTT-deficient cells post-DNA damage underscores the role of HTT in maintaining genome stability. In this study, FBXW7 knockdown alleviated G1 arrest and increased Q111 cell proliferation and improved cell proliferation and survival after DNA damage, which is consistent with the enhanced DNA repair capacity (Fig. 6). Given the critical roles of FBXW7 in both neurodevelopment and neurodegeneration, it represents a promising therapeutic target for related disorders. However, drug development targeting FBXW7 faces significant challenges due to its involvement in diverse physiological processes and the wide range of substrates it regulates, raising concerns about potential side effects. For instance, FBXW7 functions as a tumor suppressor, and its inactivation can promote tumorigenesis and resistance to certain chemotherapies [22]. Additionally, tissue-specific regulatory mechanisms and substrate selectivity complicate the design of precise therapeutics. The distinct functions of FBXW7 isoforms are not yet fully understood, although isoform-specific targeting may improve treatment specificity for neurodevelopmental and neurodegenerative impairments. Therefore, while targeting FBXW7 or its interactions with substrates holds therapeutic potential, these challenges warrant careful consideration in future drug development efforts.

Collectively, we propose that the dysregulated FBXW7 function and subsequent elevation of CHK2 protein levels driven by expanded polyQ contribute significantly to the cellular dysfunction observed in HD (Fig. 7). This dysregulation likely leads to prolonged G1/S phase arrest, increased vulnerability to DNA damage, heightened cellular death, and ultimately exacerbates the progression of HD. We suggest that the FBXW7-CHK2 axis could be a potential therapeutic target modulating DDR and improving cellular resilience in HD.

## Materials and methods

### Cell lines, DNA damage, drugs, siRNA

All cell lines were cultivated in Dulbecco’s Modified Eagle Medium (Gibco, Waltham, MA, USA) supplemented with 10% fetal bovine serum (FBS, Gibco, USA), 100 U/ml penicillin, and 100 µg/ml streptomycin (Gibco, USA). HeLa was obtained from the American Type Culture Collection (ATCC, Manassas, VA, USA). HCT116 and HCT116 FBXW7−/− cell lines were gifted by Dr Vogelstein and Dr Meredith [19]. To perform animal experiments, HD mice were used, R6/2 (The Jackson Laboratory, Bar Harbor, ME, USA) and littermate of R6/2 as a WT control. All procedures were conducted in accordance with the guidelines for the care and use of laboratory animals at Yonsei University, as approved by the Association for Assessment and Accreditation of Laboratory Animal Care. For the HD cell culture experiments, mouse striatal cell lines, WT STHdh (Q7/7) and the homozygous mutant STHdh (Q111/111) lines, were obtained from the Coriell Institute (Camden, NJ, USA). DNA damage was led by treatment of HU (Sigma, St. Louis, MJ, USA), 3 Nitropropionic acid (3NP, Sigma, USA) and gamma irradiation (IR) were used.

### RNA interference

For knockdown a target gene, cells were transfected with the indicated siRNAs using lipofectamine RNAiMAX (Invitrogen, Carlsbad, CA, USA) according to the manufacturer’s instructions for 72 h. siRNAs against human HTT (J-003737, Dhamarcon, Lafayette, CO, USA) and FBXW7 (s30663 and s30664, ThermoFisher Scientific, Waltham, MA, USA) have been used with 30 nM. As a control, negative control siRNA (D-001810) was used. The Dhamarcon (Lafayette, USA).

### Comet assay

None-damaged or DNA damaged cells were performed the comet assay by the manufacturer’s instructions (Cell Biolabs, San Diego, CA, USA). Single cell suspensions were harvested and resuspended in chilled PBS (at 1 × 10^5^ cells/mL) and mixed at a 10:1 ratio (agarose : cell), and the mixture was immediately transferred onto the top of the agarose base layer slide well. The solidified slides were lysed in alkaline lysis buffer in the dark at 4 °C for 90 min and then placed in a chilled alkaline solution in the dark at 4 °C for 30 min. Upon the completion of the reaction, the slides were electrophoresed by applying voltage to the chamber (1 volt/cm on BioRad GT mini, Hercules, California, USA) in the dark, in a cold room for 30 min. The slides were washed and stained with Green DNA dye in TE buffer. The images were obtained using a fluorescence microscope and analyzed by ImageJ (imagej.nih.gov) for % DNA contents of the tail. The comet of each cell was analyzed with the CometAnalyzer program [23].

### EdU staining for detecting cell proliferation

Cells were cultured on coverslips overnight. For EdU labeling, the culture medium was partially replaced medium resulting in a final concentration of 10 µM EdU. Cells were then incubated for 4 h at 37 °C in a 5% CO_2_ incubator to allow EdU incorporation. Following EdU incubation, the medium was removed, and cells were fixed by adding 3.7% paraformaldehyde. Fixed cells were washed twice with 3% BSA in PBS. Permeabilization was performed by adding 0.3% Triton® X-100 in PBS to each well, and cells were washed twice with 3% BSA in PBS. EdU detection was carried out using a Click-iT® EdU detection kit according to the manufacturer’s instructions (Invitrogen, Carlsbad, CA, USA). A Click-iT® reaction cocktail was prepared, and 100 µL was added to each well of a 24-well plate. After protecting from light, the reaction cocktail was removed, and cells were washed once with 3% BSA in PBS. For nuclear visualization, coverslips were mounted using Vectashield® mounting medium containing DAPI (Vectashield, USA).

### Western blotting

Harvested cells were lysed in M-per mammalian protein extraction reagent (Thermofisher, USA) supplemented with protease and phosphatase inhibitors (Protein Halts, ThermoFisher, USA) for 30 min on ice. The proteins were equally loaded and separated on a 4–15% SDS-polyacrylamide gel and transferred onto a polyvinylidene difluoride membrane (Millipore, Darmstadt, Germany). After the blocking step, each blot was probed overnight at 4 °C with specific primary antibodies as follows: γH2AX (abcam, Cambridge, MA, USA) HTT (Mab2166, Millipore, Darmstadt, Germany), HTT (1C2, Millipore, Germany), CHK1 (Cell signaling biotechnology, USA), pCHK1 (Cell signaling biotechnology, USA), CHK2 (Cell signaling biotechnology, USA), pCHK2 (Cell signaling biotechnology, USA), p-p53 (abcam, USA), Ku70 (Santa Cruz Biotechnology, USA), FBXW7 (Millipore, Darmstadt, Germany), USP28 (Proteintech, Rosemont, IL, USA), CSN6 (Santa Cruz Biotechnology, USA), and Flag (ThermoFisher, USA). Each blot was then incubated with HRP conjugated anti-mouse, or rabbit IgG antibodies (Jackson ImmunoResearch, West Grove, PA, USA) for 1 h at room temperature. For internal loading control, blots were probed with HRP-conjugated β-actin (Santa Cruz Biotechnology, USA) or HRP-vinculin (Santa Cruz Biotechnology, USA). Bands were detected by chemiluminescence (ECL, Pierce, Dallas, TX, USA) and visualized on LAS 4000 (Fujifilm, Tokyo, Japan).

### Immunocytochemistry

Cells were plated on a coverslip in a multi-well plate (3 × 10^5^ cells/ml). DNA constructs or siRNA were transfected into cells the next day. After an additional 48 h, cells were each exposed to damage for an appropriate duration. Cells were fixed in 4% PFA and permeabilized with 1% TritonX-100 in PBS. Cells were blocked in 3% BSA in PBS, and then incubated with each primary antibody overnight at 4 °C. Thereafter, each cell was incubated with fluorescence-conjugated secondary antibodies for 1 h at room temperature. In each step, cells were washed with PBS with 0.1% Tween 20 three times for 5 min. For mounting, cells on a coverslip were mounted by Vectashield containing 4′, 6-diamidino-2-phenylindole (DAPI) (Vector Laboratory, Burlingame, CA, USA). Images for each cell were captured using a Zeiss LSM 710 confocal microscope (Carl Zeiss, Jena, Germany).

### Cell cycle assay

Cells were harvested using Trypsin/EDTA, and single-cell suspensions were prepared in PBS supplemented with 0.1% BSA. Cells were washed twice by centrifugation at 300 × *g* for 5 min and then resuspended to a concentration of 5 × 10⁶ cells/ml. Aliquots of 500 µL of the cell suspension were transferred to conical tubes. Five milliliters of cold 70% ethanol was added dropwise to the tubes while gently vortexing to prevent cell aggregation during fixation. Cells were fixed with 70% EtOH at 4 °C. Fixed cells were then washed twice, and cell pellets were resuspended in 1 ml of propidium iodide staining solution (20 µg/ml) (Molecular Probes, Eugene, OR, USA). RNase A stock solution (50 µL from a 100 µg/mL stock) (Molecular Probes, USA) was added to achieve a final concentration of 0.5 µg/ml, and the samples were incubated for 4 h at 4 °C. The stained samples were analyzed with FACS (BD FACSymphony A5 SE, BD Biosciences, San Jose, CA, USA).

### Real time quantitative RT-PCR

Total RNA from cells was extracted using the HiGene Total RNA Prep Kit (BIOFACT Co. Ltd., Daejeon, Korea). Reverse transcription quantitative PCR (RT-qPCR) was performed using the One-Step SYBR PrimeScript RT-PCR Kit (TaKaRa Bio Inc., Shiga, Japan) on a QS3 real-time PCR system (Applied Biosystems, Waltham, MA, USA). PCR amplification consisted of 40 cycles, with denaturation at 95 °C for 5 s, followed by annealing and extension at 60 °C for 60 s. Each sample was run in triplicate, including a no-template control, and the results were subsequently analyzed. Differential expression levels were analyzed using the 2^−∆∆Ct^ method and expressed as relative quantity. The primer sequences for FBXW7 (mouse), 5′-CGAGACTTCATCTCCTTGCTTCC-3′, 5′-CCAGAGAAGGTTATCCTCAGCC-3′; HTT (human) 5′-CTCTGGTGTCAGATACTGCTGC-3′, 5′-CTCCTCTTCTCCAGACATCTGG-3′; ß-actin (mouse), 5′-CCTGAACCCTAAGGCCAACC-3′, 5’ATGGCGTGAGGGAGAGCATA-3′; ß-actin (human), ß -actin (human), 5′-CACCATTGGCAATGAGCGGTTC-3′, 5′-AGGTCTTTGCGGATGTCCACGT-3′.

### Proximity ligation assay (PLA)

PLA was performed according to the manufacture’s protocol. Cells plated in 8-well slide were fixed with FA and blocking with blocking solution included in the Duolink PLA kit (Sigma, USA). Each primary antibody was added to the cells and incubated. Cells were incubated with PLA probe, ligated with PLA ligase, and then amplified with PLA polymerase in amplification buffer including appropriate fluorescence red or green. Slides were washed and mounted with in situ mounting medium with DAPI. Cells were analyzed under confocal microscope LSM710 (Carl Zeiss, Germany).

### Colony formation assay

Cells were plated 1 × 10^3^–5 × 10^3^ cells per well into a 6-well plate. Cells were treated with siFBXW7 for 48 h and additional DMSO or HU (1 mM) for 24 h. And then cells were replaced with fresh media and incubated until colonies were observed. Colonies fixed with 4% PFA were stained with 0.05% Crystal violet. Each colony in a well was counted all. Proliferating efficiency was presented as a percentage of colony number per originally plated cell number.

### Statistical analysis

Data are presented as mean ± standard deviation and analyzed using GraphPad Prism software (version 8.0). Statistical analyses were performed using unpaired t-test and one-way ANOVA. Differences were statistically significant at *p < 0.01, and **p < 0.001

## Supplementary information

Supplementary Figure 1.

## Data Availability

All data and materials that support the findings of this study are available.

## References

[1] Maiuri T, Suart CE, Hung CLK, Graham KJ, Barba Bazan CA, Truant R. DNA damage repair in Huntington’s disease and other neurodegenerative diseases. Neurotherapeutics. 2019;16:948–56. 10.1007/s13311-019-00768-7. 31364066 10.1007/s13311-019-00768-7 PMC6985310

[2] Antikainen H, Driscoll M, Haspel G, Dobrowolski R. TOR-mediated regulation of metabolism in aging. Aging Cell. 2017;16:1219–33. 10.1111/acel.12689. 28971552 10.1111/acel.12689 PMC5676073

[3] Lee JM, Huang Y, Orth M, Gillis T, Siciliano J, Hong E, et al. Genetic modifiers of Huntington disease differentially influence motor and cognitive domains. Am J Hum Genet. 2022;109:885–99. 10.1016/j.ajhg.2022.03.004. 35325614 10.1016/j.ajhg.2022.03.004 PMC9118113

[4] Massey TH, Jones L. The central role of DNA damage and repair in CAG repeat diseases. Dis Model Mech. 2018;11. 10.1242/dmm.031930. 10.1242/dmm.031930 PMC5818082 29419417

[5] Maiuri T, Mocle AJ, Hung CL, Xia J, van Roon-Mom WM, Truant R. Huntingtin is a scaffolding protein in the ATM oxidative DNA damage response complex. Hum Mol Genet. 2017;26:395–406. 10.1093/hmg/ddw395. 28017939 10.1093/hmg/ddw395

[6] Atha DH, Reipa V. Standards for quantitative measurement of DNA damage in mammalian cells. Int J Mol Sci. 2023;24. 10.3390/ijms24065427. 10.3390/ijms24065427 PMC10051712 36982502

[7] Bohgaki M, Hakem A, Halaby MJ, Bohgaki T, Li Q, Bissey PA, et al. The E3 ligase PIRH2 polyubiquitylates CHK2 and regulates its turnover. Cell Death Differ. 2013;20:812–22. 10.1038/cdd.2013.7. 23449389 10.1038/cdd.2013.7 PMC3647240

[8] Kisielewska M, Filipski M, Sebastianka K, Karas D, Molik K, Choromanska A. Investigation into the neuroprotective and therapeutic potential of plant-derived Chk2 Inhibitors. Int J Mol Sci. 2024;25. 10.3390/ijms25147725. 10.3390/ijms25147725 PMC11277127 39062967

[9] Gomez-Pastor R, Burchfiel ET, Neef DW, Jaeger AM, Cabiscol E, McKinstry SU, et al. Abnormal degradation of the neuronal stress-protective transcription factor HSF1 in Huntington’s disease. Nat Commun. 2017;8:14405. 10.1038/ncomms14405. 28194040 10.1038/ncomms14405 PMC5316841

[10] Yang Y, Zhou X, Liu X, Song R, Gao Y, Wang S. Implications of FBXW7 in neurodevelopment and neurodegeneration: molecular mechanisms and therapeutic potential. Front Cell Neurosci. 2021;15:736008. 10.3389/fncel.2021.736008. 34512273 10.3389/fncel.2021.736008 PMC8424092

[11] Fan J, Bellon M, Ju M, Zhao L, Wei M, Fu L, et al. Clinical significance of FBXW7 loss of function in human cancers. Mol Cancer. 2022;21:87. 10.1186/s12943-022-01548-2. 35346215 10.1186/s12943-022-01548-2 PMC8962602

[12] Wang L, Chen R, Li G, Wang Z, Liu J, Liang Y, et al. FBW7 mediates senescence and pulmonary fibrosis through telomere uncapping. Cell Metab. 2020;32:860–77. 10.1016/j.cmet.2020.10.004. 33086033 10.1016/j.cmet.2020.10.004

[13] Joshi DC, Chavan MB, Gurow K, Gupta M, Dhaliwal JS, Ming LC. The role of mitochondrial dysfunction in Huntington’s disease: implications for therapeutic targeting. Biomed Pharmacother. 2025;183:117827. 10.1016/j.biopha.2025.117827. 39854819 10.1016/j.biopha.2025.117827

[14] Liu C, Fu Z, Wu S, Wang X, Zhang S, Chu C, et al. Mitochondrial HSF1 triggers mitochondrial dysfunction and neurodegeneration in Huntington’s disease. EMBO Mol Med. 2022;14:e15851. 10.15252/emmm.202215851. 35670111 10.15252/emmm.202215851 PMC9260212

[15] Lu XH, Mattis VB, Wang N, Al-Ramahi I, van den Berg N, Fratantoni SA, et al. Targeting ATM ameliorates mutant Huntingtin toxicity in cell and animal models of Huntington’s disease. Sci Transl Med. 2014;6:268ra178 10.1126/scitranslmed.3010523. 25540325 10.1126/scitranslmed.3010523

[16] Lu H, Saha J, Beckmann PJ, Hendrickson EA, Davis AJ. DNA-PKcs promotes chromatin decondensation to facilitate initiation of the DNA damage response. Nucleic Acids Res. 2019;47:9467–79. 10.1093/nar/gkz694. 31396623 10.1093/nar/gkz694 PMC6765147

[17] Kopper F, Bierwirth C, Schon M, Kunze M, Elvers I, Kranz D, et al. Damage-induced DNA replication stalling relies on MAPK-activated protein kinase 2 activity. Proc Natl Acad Sci USA. 2013;110:16856–61. 10.1073/pnas.1304355110. 24082115 10.1073/pnas.1304355110 PMC3801042

[18] Gagou ME, Zuazua-Villar P, Meuth M. Enhanced H2AX phosphorylation, DNA replication fork arrest, and cell death in the absence of Chk1. Mol Biol Cell. 2010;21:739–52. 10.1091/mbc.e09-07-0618. 20053681 10.1091/mbc.E09-07-0618 PMC2828961

[19] Rajagopalan H, Jallepalli PV, Rago C, Velculescu VE, Kinzler KW, Vogelstein B, et al. Inactivation of hCDC4 can cause chromosomal instability. Nature. 2004;428:77–81. 10.1038/nature02313. 14999283 10.1038/nature02313

[20] Choi SH, Cho K, Kim ES, Yoo HY. Proline-serine-threonine-repeat region of MDC1 mediates Chk1 phosphorylation and the DNA double-strand break repair. Int J. Biochem Cell Biol. 2022;143:106152. 10.1016/j.biocel.2021.106152. 34974185 10.1016/j.biocel.2021.106152

[21] Kharat SS, Tripathi V, Damodaran AP, Priyadarshini R, Chandra S, Tikoo S, et al. Mitotic phosphorylation of Bloom helicase at Thr182 is required for its proteasomal degradation and maintenance of chromosomal stability. Oncogene. 2016;35:1025–38. 10.1038/onc.2015.157. 26028025 10.1038/onc.2015.157

[22] Yumimoto K, Nakayama KI. Recent insight into the role of FBXW7 as a tumor suppressor. Semin Cancer Biol. 2020;67:1–15. 10.1016/j.semcancer.2020.02.017. 32113998 10.1016/j.semcancer.2020.02.017

[23] Beleon A, Pignatta S, Arienti C, Carbonaro A, Horvath P, Martinelli G, et al. CometAnalyser: a user-friendly, open-source deep-learning microscopy tool for quantitative comet assay analysis. Comput Struct Biotechnol J. 2022;20:4122–30. 10.1016/j.csbj.2022.07.053. 36016714 10.1016/j.csbj.2022.07.053 PMC9385450

