# Supplementary material for: FBXW7-mediated CHK2 regulation modulates DNA damage response and cellular stability in Huntington’s disease
Source: Cell Death Discov. 2025 Nov 3;11:499. doi: 10.1038/s41420-025-02798-x (PMC12583571; doi:10.1038/s41420-025-02798-x)

Supplementary Figure 1. Uncropped Western blot images.

The uncropped Western blot (WB) images corresponding to the cropped bands presented in the main figures are shown. All WB data were acquired using the LAS imaging system. Molecular weight markers are indicated. These uncropped blots are provided to ensure transparency and allow verification of the integrity of the data presented in Figures 1, 2, 3, 4, and 5.

Figure 1

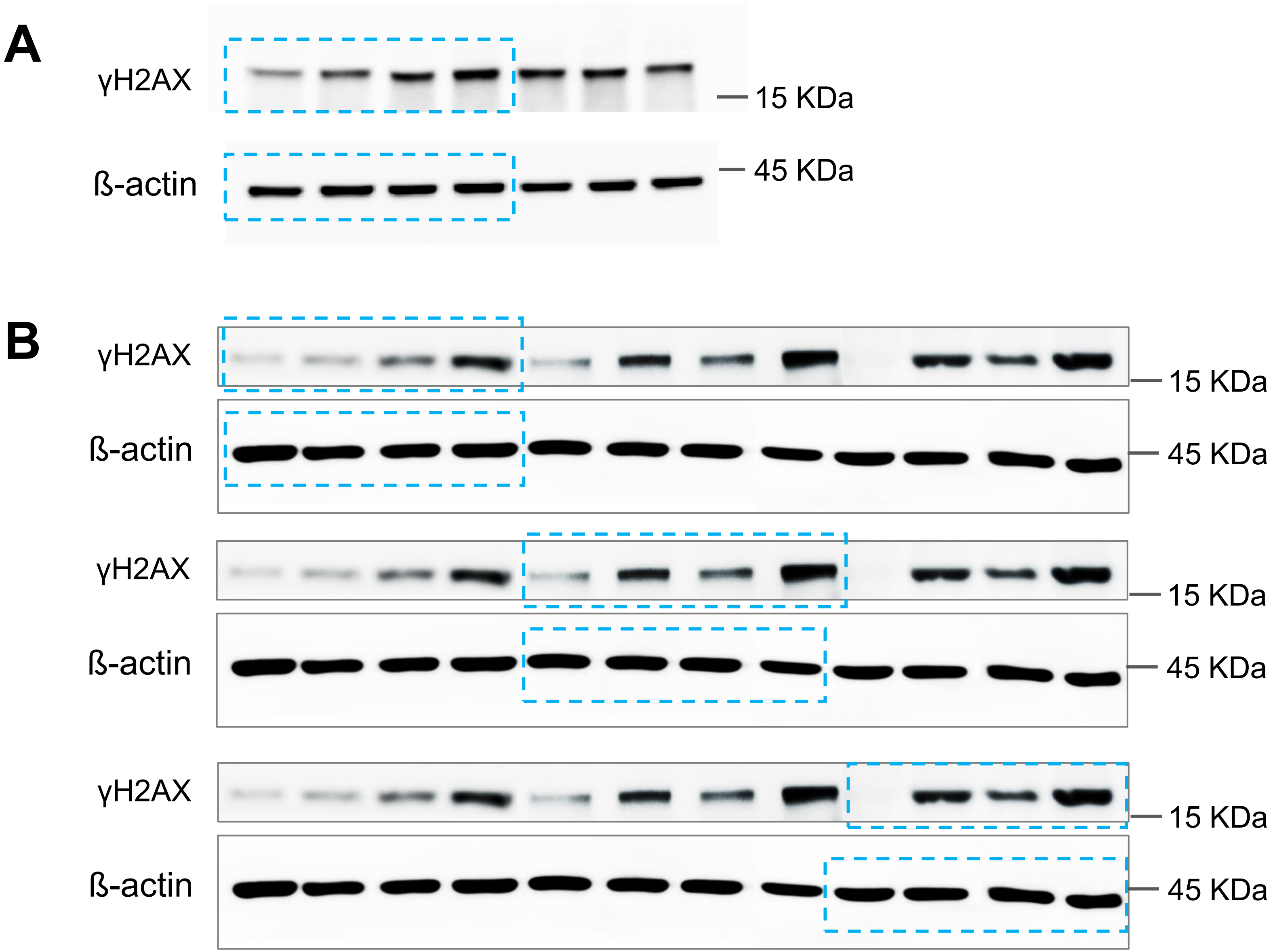

A
γH2AX
15 KDa
ß-actin
45 KDa
B
γH2AX
15 KDa
ß-actin
45 KDa
γH2AX
15 KDa
ß-actin
45 KDa
γH2AX
15 KDa
ß-actin
45 KDa

Figure 2

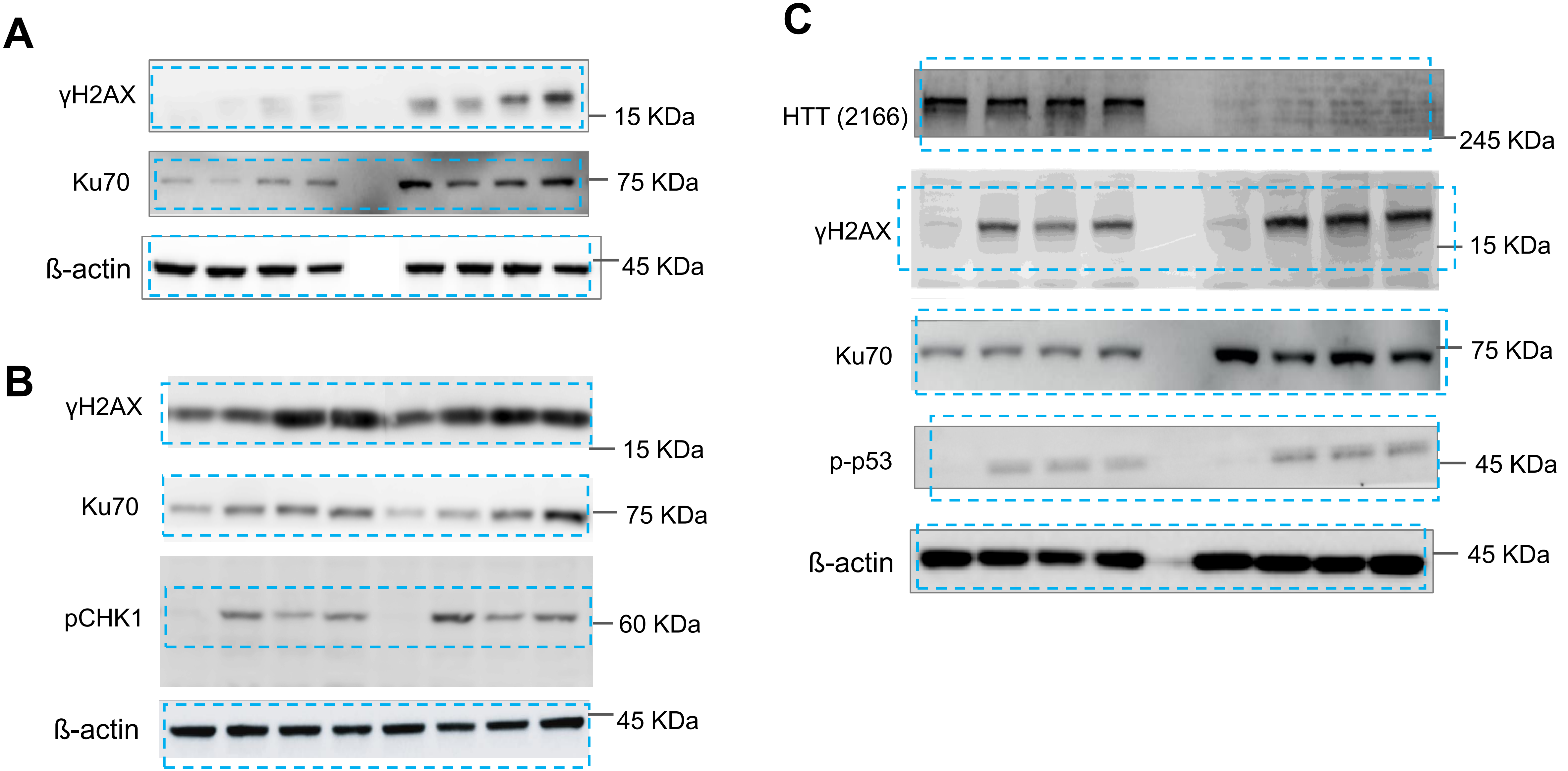
A
γH2AX
15 KDa
Ku70
75 KDa
ß-actin
45 KDa
B
γH2AX
15 KDa
Ku70
75 KDa
pCHK1
60 KDa
ß-actin
45 KDa
C
HTT (2166)
245 KDa
γH2AX
15 KDa
Ku70
75 KDa
p-p53
45 KDa
ß-actin
45 KDa

Figure 3

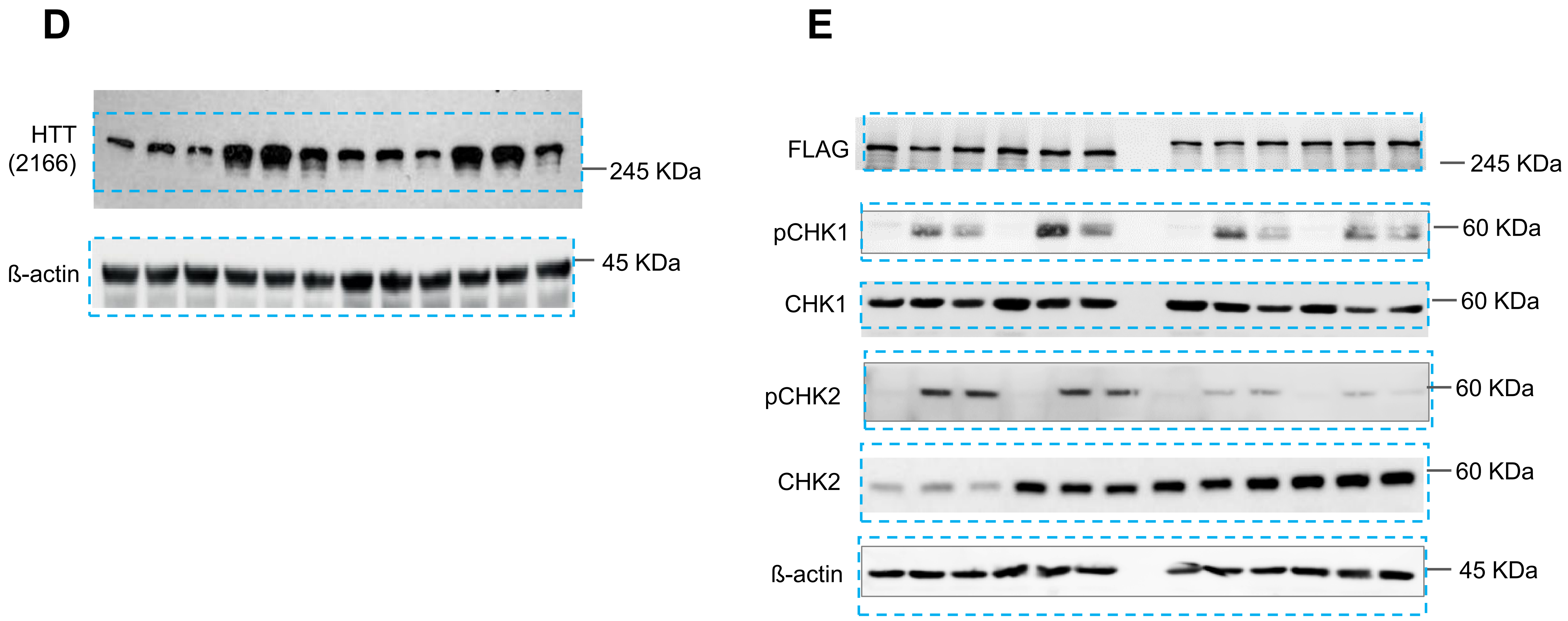
D
HTT
(2166)
245 KDa
ß-actin
45 KDa
E
FLAG
245 KDa
pCHK1
60 KDa
CHK1
60 KDa
pCHK2
60 KDa
CHK2
60 KDa
ß-actin
45 KDa

Figure 4

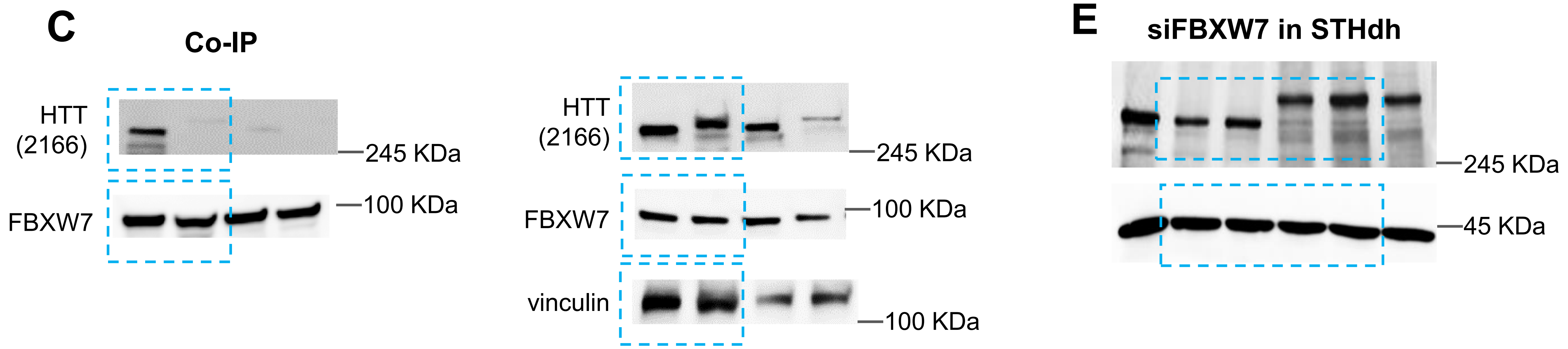
C
Co-IP
HTT
(2166)
245 KDa
FBXW7
100 KDa
HTT
(2166)
245 KDa
FBXW7
100 KDa
vinculin
100 KDa
E
siFBXW7 in STHdh
245 KDa
45 KDa

Figure 4

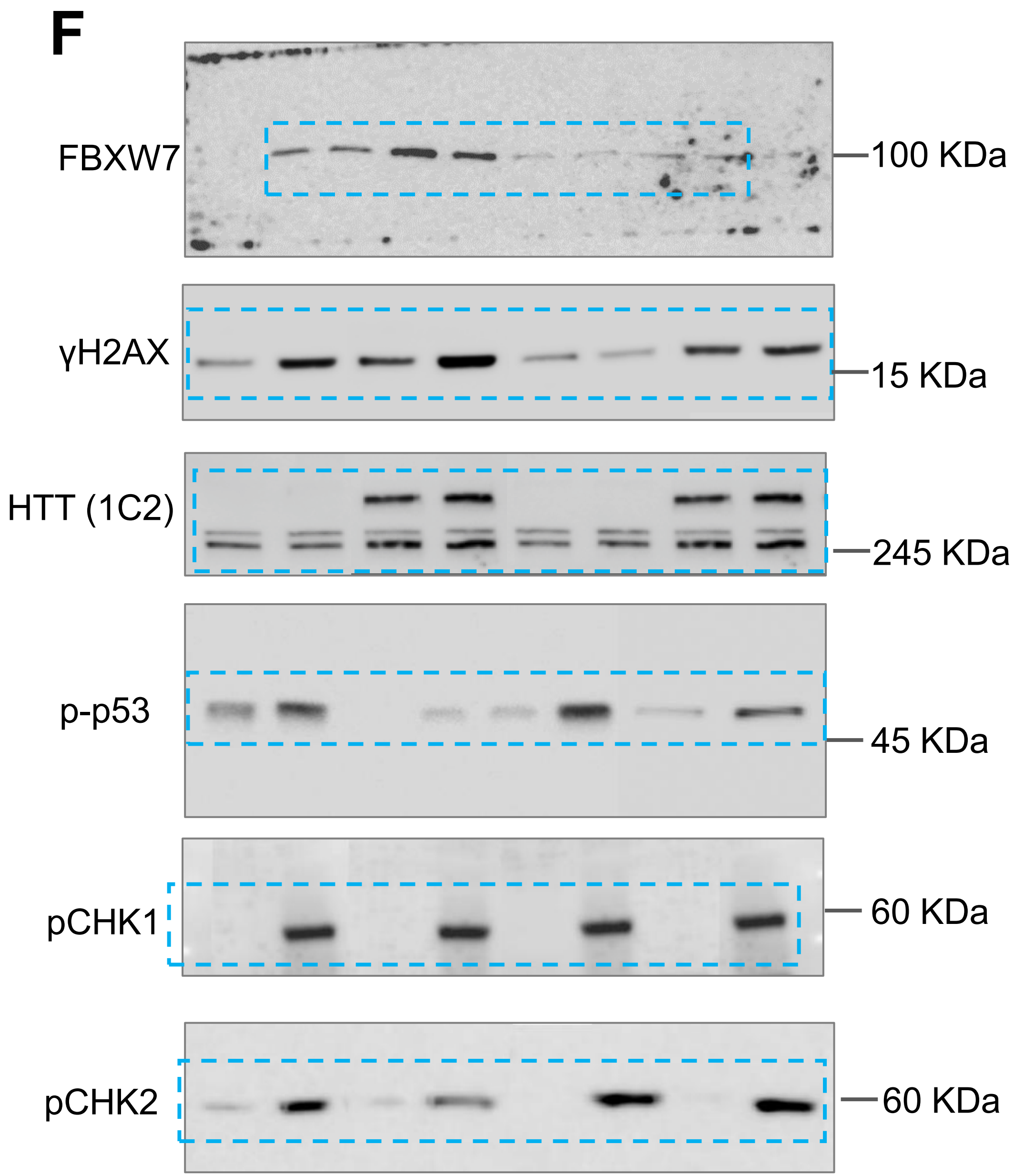

F
FBXW7
100 KDa
γH2AX
15 KDa
HTT (1C2)
245 KDa
p-p53
45 KDa
pCHK1
60 KDa
pCHK2
60 KDa

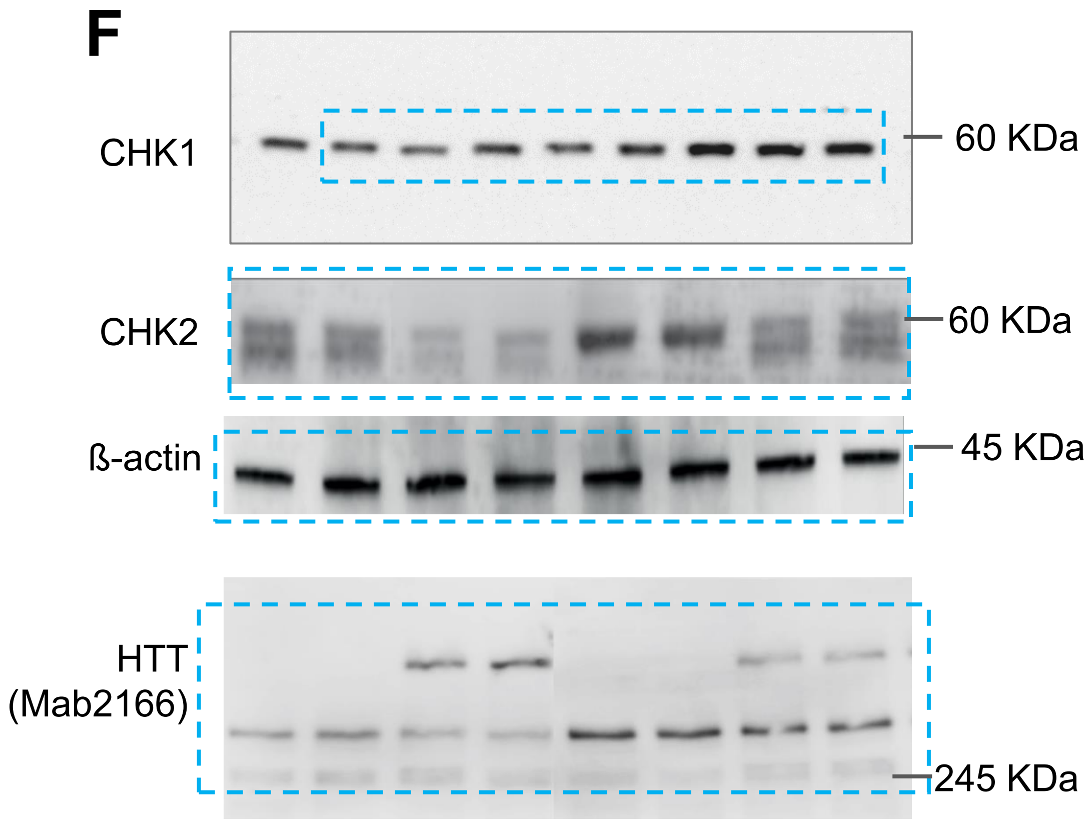

F
CHK1
60 KDa
CHK2
60 KDa
ß-actin
45 KDa
HTT
(Mab2166)
245 KDa

Figure 5

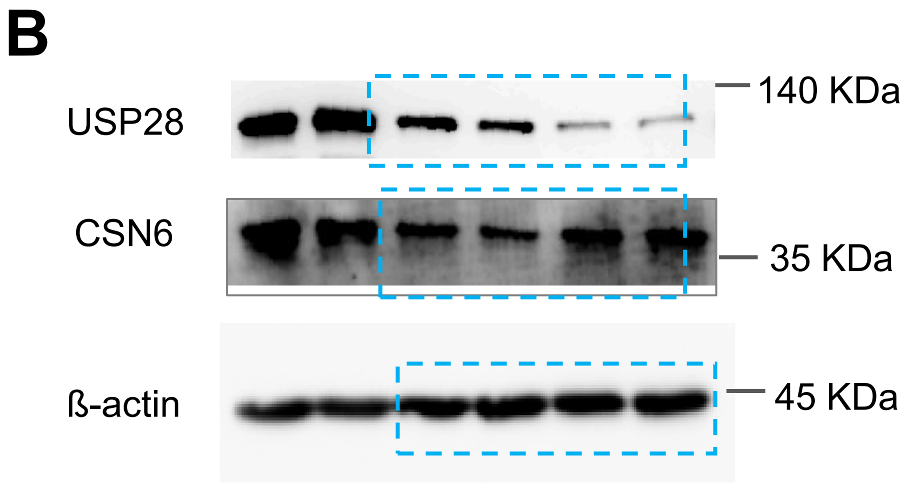

Supplement: Supplementary file 1 — Supplementary Figure 1. [file 41420_2025_2798_MOESM1_ESM.pdf]
